# Genetic Characterization and Variation of African Swine Fever Virus China/GD/2019 Strain in Domestic Pigs

**DOI:** 10.3390/pathogens11010097

**Published:** 2022-01-14

**Authors:** Xun Wang, Xiaoying Wang, Xiaoxiao Zhang, Sheng He, Yaosheng Chen, Xiaohong Liu, Chunhe Guo

**Affiliations:** State Key Laboratory of Biocontrol, School of Life Sciences, Sun Yat-sen University, North Third Road, Guangzhou Higher Education Mega Center, Guangzhou 510006, China; wangx597@mail3.sysu.edu.cn (X.W.); wangxy33@mail.sysu.edu.cn (X.W.); zhangxx55@mail2.sysu.edu.cn (X.Z.); hesh56@mail2.sysu.edu.cn (S.H.); chyaosh@mail.sysu.edu.cn (Y.C.); liuxh8@mail.sysu.edu.cn (X.L.)

**Keywords:** African swine fever virus, evolution, mutation, core gene, pan gene, phylogenetic tree

## Abstract

African swine fever (ASF) was first introduced into Northern China in 2018 and has spread through China since then. Here, we extracted the viral DNA from the blood samples from an ASF outbreak farm in Guangdong province, China and sequenced the whole genome. We assembled the full length genomic sequence of this strain, named China/GD/2019. The whole genome was 188,642 bp long (terminal inverted repeats and loops were not sequenced), encoding 175 open reading frames (ORF). The China/GD/2019 strain belonged to p72 genotype II and p54 genotype IIa. Phylogenetic analysis relationships based on single nucleotide polymorphisms (SNPs) also demonstrated that it grouped into genotype II. A certain number of ORFs mainly belonging to multigene families (MGFs) were absent in the China/GD/2019 strain in comparison to the China/ASFV/SY-18 strain. A deletion of approximately 1 kb was found in the China/GD/2019 genome which was located at the EP153R and EP402R genes in comparison to the China/2018/AnhuiXCGQ strain. We revealed a synonymous mutation site at gene F317L and a non-synonymous mutation site at gene MGF_360-6L in China/GD/2019 comparing to three known Chinese strains. Pair-wise comparison revealed 165 SNP sites in MGF_360-1L between Estonia 2014 and the China/GD/2019 strain. Comparing to China/GD/2019, we revealed a base deletion located at gene D1133L in China/Pig/HLJ/2018 and China/DB/LN/2018, which results in a frameshift mutation to alter the encoding protein. Our findings indicate that China/GD/2019 is a new variant with certain deletions and mutations. This study deepens our understanding of the genomic diversity and genetic variation of ASFV.

## 1. Introduction

African swine fever (ASF) is a highly pathogenic infectious disease caused by African swine fever virus (ASFV) [1,2]. Since the ASFV genome is complex and encodes many genes that have different functions [3], it is difficult to develop vaccines and drugs against ASFV infection [4,5,6]. Since the disease was first introduced into China in 2018, it has spread rapidly and has a tendency to sweep the whole country it is present in [7,8,9,10].

With huge molecular weight and linear double-stranded DNA, ASFV is the only member of *Asfvirus* genus within the *Asfarviridae* virus family [3,11]. The genome of the virus ranges in size from 170 to 193 kb, containing 150–167 open reading frames (ORF), of which the function of one third is unknown. It consists of a conserved central region and a variable region at both ends (containing five multigene families, MGFs) [3,12]. Most of the variations among ASFV genomes are due to the presence of different numbers of MGF genes in the left or right variable regions (LVR and RVR) [13,14,15]. MGFs are characteristic of the virus; five families have been recognized—MGF 100, 110, 300, 360 and 505/530 [16,17]—and the function of many is still unknown. With the spread of ASFV for so many years, the virus has already had a lot of variations and divergences in the genome [18]. Fortunately, in recent years, complete genome sequences from strains of different origins have become more easily available along with comparative analyses [19,20].

No study on the characterizations of the complete genome of strains responsible for ASF outbreaks in Guangdong province in Southern China is available. Using the next generation sequencing technique, the complete genome sequence of the ASFV China/GD/2019 strain was assembled. Using phylogenetic analysis based on full length p72 and p54 genes, the China/GD/2019 strain clustered into genotype II. We used phylogenetic analysis to identify the different origin and genotype strain relationships based on SNPs, and the China/GD/2019 strain clustered with genotype II strains and showed high similarity with Estonia 2014 on encoding genes. A detailed genomic comparison of the China/GD/2019 strain with related p72 genotype II isolates on encoding genes and SNPs was conducted. We compared China/GD/2019’s genome with China/2018/AnhuiXCGQ by comparative genomic analysis. We found a deletion of approximately 1 kb in the China/GD/2019 genome which was located at the EP153R and EP402R genes. According to SNP/InDel analysis, a large number of mutations were found between Estonia 2014 and China/GD/2019. A synonymous mutation site at gene F317L and a non-synonymous mutation site at MGF_360-6L were detected in the China/GD/2019 strain; we also found a base deletion at gene D1133L in the China/Pig/HLJ/2018 and China/DB/LN/2018 strains. By comparing core and pan genes, we found that 14 MGF members were absent from the China/GD/2019 strain in the MGF regions (especially 360 and 110 multigenes) in comparison to the China/ASFV/SY-18 isolate. Other genes whose function is unknown were also found to be missing compared to the China/2018/SY-18 isolate. It is of no doubt that China/GD/2019 is a new member of ASFV family with certain deletions and mutations. This study of genome characteristics of ASFV is of great significance for the source tracing and prevention and control of ASFV.

## 2. Results

### 2.1. Complete Genome Sequence of ASFV China/GD/2019 Strain

The complete genome sequence of the ASFV China/GD/2019 strain was 188,642 bp in length, not including terminal inverted repeats and cross links. The final assembly of the China/GD/2019 strain genome was accomplished from a reference-based alignment consisting of 1.925 Gb mapped reads with an average depth 100×. The genome of this strain is considerably smaller than that of other Chinese ASFV isolates. We used three databases to predict gene functions: Gene Ontology (GO), Kyoto Encyclopedia of Genes and Genomes (KEGG), and Swiss-Prot. We identified 175 ORFs, the function of the ASFV China/GD/2019 strain encoding genes is involved in virus assembly, enzymes, extracellular region parts, and viral reproduction.

### 2.2. Phylogenetic Analysis of Full Length p72 (B646L) and p54 (E183L) Genes

**Figure 1:**
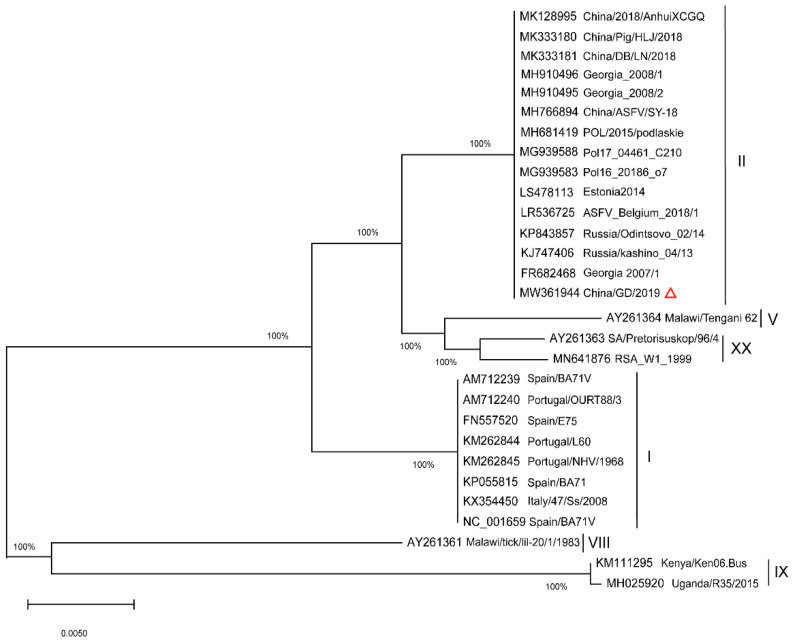
Phylogenetic relationships of the full length p72 (B646L) gene of China/GD/2019 strain and 28 publicly available ASFV isolates. The full length of B646L gene relative to the 28 known p72 genotype (labeled I-XX) strains and China/GD/2019 strain were used for analysis. The evolutionary relationships were performed by TreeBeST software using the neighbor-joining method. The GenBank accession numbers of strains used in the study are indicated. The characteristic virus strain in this study is indicated in a red triangle. The scale bar indicates numbers of substitutions per site.

To determine the genetic relationship between the China/GD/2019 strain and other previously identified ASFV genotype (I-XX p72) isolates listed in Table 1 (for which p72 sequences were available), the ASFV p72 gene phylogenetic tree was constructed (Figure 1).

**Figure 2:**
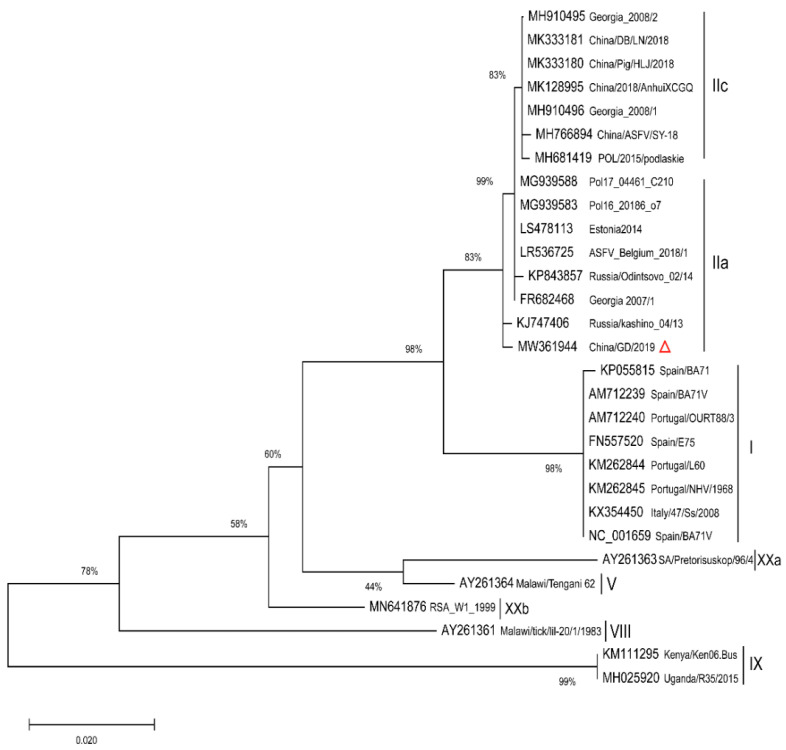
Phylogenetic relationships of the full length p54 (E183L) gene of China/GD/2019 and 28 publicly available ASFV isolates. The full length of E183L gene relative to the 28 known p72 genotype (labeled I-XX) strains and the China/GD/2019 strain were used for analysis. The evolutionary relationships were performed by TreeBeST software using the neighbor-joining method. The GenBank accession numbers of strains used in the study are indicated. The characteristic virus strain in this study is indicated in a red triangle. The scale bar indicates numbers of substitutions per site.

A phylogenetic tree based on the full length p72 (B646L) sequence alignment indicated that the new ASFV strain belongs to genotype II and is similar to those from Chinese reporting in recent years; it is certain that all the ASFVs circulating in China belong to genotype II. Nucleotide sequence comparisons by the Basic Local Alignment Search Tool (BLAST) revealed that the p72 sequence of China/GD/2019 was 100% identical to other Chinese isolates. Furthermore, a p54 NJ tree was constructed using the full length p54 sequences (Figure 2) from the strains in Table 1 (for which p54 sequences were available). Undoubtedly, the p54 genetic tree showed that p72 genotype II strains were separated into similar genotype II, consistent with the p72 evolutionary relationship. The China/2018/AnhuiXCGQ, China/Pig/HLJ/2018 and China/DB/LN/2018 strains belong to p54 genotype IIc, while China/GD/2019 was not clustered within the identification of the previously reported four Chinese genotype II variants, and was closely related to Georgia 2007/1 (genotype IIa). These data indicate that the strain in the Southern China could be diverse, and the ASFV strains in China in 2019 are related to that of China in 2018 and Eastern Europe.

### 2.3. Core Genes and Specific Genes Analysis

To understand the difference of coding genes in different strains more intuitively, we selected previously reported ASFV isolates, and combined with the China/GD/2019 strain to perform core and pan genes analysis. The number of ASFV pan genes may expand with each added genome which contributes to understand the evolution relationship. Conversely, the number of ASFV core genes may decline as each genome increases, most of which are critical to the ASFV survival.

We obtained 116 core genes and 249 pan genes in different samples. The 116 core genes identity of 28 ASFV strains with China/GD/2019 is listed in Supplementary Table S1.

We performed ORFs identity comparison of 28 ASFV strains with China/GD/2019, which shows the missing coding proteins. These deleted genes were located at MGF 505, MGF 110, MGF 300, MGF 100, and MGF 360. Deleted MGF regions in the China/GD/2019 strain were mainly located at MGF 360 and 110, with few of them located at MGF 300, 100 and 505.

Since China/GD/2019 belongs to genotype II, and is similar to those strains from China and Eastern Europe reported in recent years, we compared China/GD/2019 with the Russia/Kashino_04/13 strain. We found 133 identical ORFs and 23 ORFs sharing 90.8–99.8% sequence identity (Supplementary Table S1). The changed ORFs included IAP-like protein (A224L), and five members of MGF (MGF_360-6L, MGF_360-13L, MGF_505-5R, and MGF_505-10R). Quite a few ORFs (X69R, A125L, A528, C122R and MGF_505-4R) with unknown function were missing in the Russia/Kashino_04/13 genome. Comparing the ORFs of China/GD/2019 with China/2018/AnhuiXCGQ, 144 identical ORFs and 14 ORFs sharing 90.8–99.9% sequence identity were identified. A125L was missing in China/2018/AnhuiXCGQ. Comparing the ORFs of China/GD/2019 with those of the Georgia 2007/1 strain revealed 33 ORFs sharing 90.8–99.8% sequence identity. The changed ORFs included DNA ligase (NP419L), DNA polymerase beta-like protein (O174L), five members of MGF (MGF_300-2R, MGF_300-4L, MGF_360-6L, MGF_360-8L, and MGF_505-11L), and unknown proteins (I267L and G1211R). Five members of MGF (MGF_360-7L, MGF_360-17R, MGF_360-21R, MGF_505-4R and MGF_505-6R) were missing in the Georgia 2007/1 strain.

Comparing China/GD/2019 with the Estonia 2014 strain, 130 identical ORFs and 14 ORFs sharing 90.8–99.9% sequence identity were identified. The changed ORFs included Helicase (A859L) and p30(CP204L). A total of 19 members of MGFs, L356L, L60L, L83L, Envelope protein p22 and several unknown function proteins were missing in the Estonia 2014 genome. Because Spain/BA71V/1971 was the first isolated Vero-cell adapted strain, which belongs to p72 genotype I, we compared the ORFs of China/GD/2019 with Spain/BA71V/1971. Only 25 identical ORFs and 95 ORFs sharing 90.6–99.9% sequence identity were identified (Supplementary Table S1). We also compared the China/GD/2019 genome with the China/2018/AnhuiXCGQ strain by collinearity analysis and found a deletion of approximately 1kb in China/GD/2019 which was located at EP153R and EP402R genes (Figure S1).

### 2.4. Phylogenetic Analysis of the SNP

**Figure 3:**
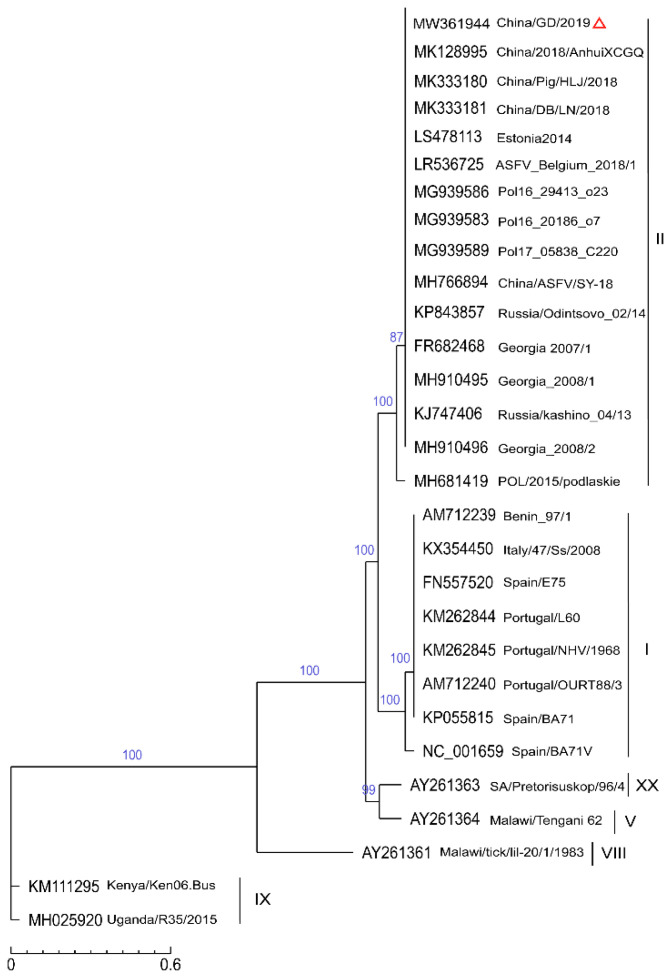
Phylogenetic relationships of the SNPs of China/GD/2019 and 28 publicly available ASFVs. The SNPs distribution relative to the 28 known p72 genotype (labeled I-XX) isolates and the China/GD/2019 strain were used for analysis. The evolutionary relationships were performed by TreeBeST software using the neighbor-joining method. The characteristic virus strain in this study is indicated in a red triangle. The scale bar indicates numbers of substitutions per site.

SNP is the most common evolutionary form of genomic variation. ASFV has a huge genome and highly susceptible to mutation, so SNPs may be either in the gene sequence or in the noncoding sequence outside the gene [3]. Based on SNPs from the complete genome level, we explored the correlation between China/GD/2019 and other different ASFV strains, and we also selected previously identified ASFV strains listed in Table 1. As shown in Figure 3, most of them belong to genotype II and all the 29 ASFV strains were also grouped into five main branches. At the top, China/GD/2019 was most closely related with three other Chinese strains (MK128995, MK333180, and MK333181), but was relatively distant to the first isolated Chinese strain (MK766894). Georgia 2007/1, Belgium_2018/1 and Estonia 2014 were also in a cluster with China/GD/2019 at high credibility. Strains from Poland (Pol16_20186_o7, Pol16_29413_o23, and Pol17_05838_C220) and Russia/Odintsovo_02/14 showed similarity to the China/GD/2019 strain in SNPs distribution. These data show that different strains belonging to the same genotype have similar SNPs and that strains with highly similar encoding genes also have similarity in SNPs distribution.

### 2.5. SNP/InDel Analysis of China/GD/2019 Strain

Through the above evolutionary tree analysis, we focused on the relative variation and similarity between this strain with other p72 genotype II isolates, so that we can learn more about the variation and characteristics of ASFV transmission in China. Based on the above phylogenetic tree relationship, we selected eight strains that were relatively similar to China/GD/2019. The SNP statistics results revealed a large number of SNPs that were discovered between China/GD/2019 and Estonia 2014 (Supplementary Table S2). About 165 SNPs were located at MGF_360-1La and MGF_360-1Lb, including 1 initial codon nonsynonymous mutations, 2 premature_stop, 63 synonymous, 102 nonsynonymous, and 4 intergenic. However, the effects of these variations cannot be determined. Comparing China/GD/2019 with the other seven strains, only a few mutations were found (Table 2). The SNPs distribution of seven ASFV strains with China/GD/2019 is listed in Supplementary Table S3.

We revealed one synonymous site, one nonsynonymous site, and four intergenic sites in China/GD/2019 comparing to China/Pig/HLJ/2018 and China/DB/LN/2018. China/GD/2019 has a synonymous mutation site at gene F317L, which A changes to T. Among Chinese ASFV strains, China/GD/2019 has a non-synonymous mutation site at MGF_360-6L, which changes nucleotide N (“N” means that any of the four nucleotides can be in this position) to T (Table 3).

There are other non-synonymous mutation sites located at genes K145R, E199L, MGF 505, MGF 360 and E184L, when comparing with other four strains from Poland and Belgium. Base substitutions affect the encoding protein only by changing the encoding amino acid, whereas insertion and deletion have the greatest impact on the genome. Therefore, we used LASTZ software to detect small fragment InDel with a length of less than 50 bp by comparing China/GD/2019 with the seven other related ASFV genotype II strains. InDel analysis results showed a base deletion in China/Pig/HLJ/2018 and China/DB/LN/2018, which was located at gene D1133L, causing frameshift mutation, and changing the encoding amino acid and protein structure.

## 3. Discussion

Since the first outbreak of ASFV in China in 2018, the pig breeding industry, especially the basic production capacity, has been affected. The present study investigated the molecular characterization of ASFV strains that occurred in 2019 in Guangdong province, Southern China. Genetic analysis showed that the ASF outbreaks in Southern China were caused by genotype II ASFV, which was highly similar to the other Chinese strains and related Eastern European (Russia and Poland) genotype II strains. This study verified that the China/GD/2019 isolate may be derived from an introduction of ASFV strains circulating in Eastern Europe [21,22]. 

The phylogenetic tree based on all available nucleotide sequences of the ASFV complete genomes indicated that full length p72 (B646L) and p54 (E183L) were similar to that of SNPs. However, further phylogenetic analysis is necessary to ascertain this relationship [23]. The key finding from our study is that p72 ASFV phylogenetic analysis genotyping results can be coordinated with other phylogenetic analysis methods. The p72 genotype II viruses are separated into genotype IIa and Iic in the p54 phylogenetic tree, suggesting that they are phylogenetically closely related, and it is clear from the latter phylogenetic tree that they do not form a monophyletic lineage. However, although the genome sequence of the ASFV strain in Guangdong province of China showed high similarity to those of recently isolated ASFV strains from China and Eastern Europe, the specific source of this strain remains unclear, probably due to the limited sequence information obtained in this study.

Comparing the genome sequence of the China/GD/2019 strain with those of Chinese and related Eastern European virulent p72 genotype II strains showed a range of 9–165 mutation sites along the genome sequences. Small numbers of SNPs have been found among Chinese strains. ASFV major structural proteins and some reported virulence factors such as MGF 360-4L, 11L, 12L, and MGF 505-1R did not contain any genetic mutations [24,25,26,27]. Furthermore, several genes were affected by point mutations, including K145R, E199L, MGF 505, MGF 360 and E184L. A total of 165 variable sites were found at MGF_360-1L between China/GD/2019 and Estonia 2014, a long time may be required to result in such huge difference.

It is suggested that the variation of ASFV in China does not simply depend on the replacement of a few or even dozens of bases, but is accompanied by the insertion or deletion of small or large fragments [28]. By comparing to the China/GD/2019 strain, a deletion region was checked at gene D1133L simultaneously in China/Pig/HLJ/2018 and China/DB/LN/2018. According to previous analyses, the insertion/deletion may be attributed much to the homologous recombination [29,30,31]; we thought these could be a variation of the ASFV as it spread in China, but the effects on the infectivity and virulence of the virus is unknown.

Most of ASFV genome variations result from gain or loss of genes in the MGFs [32]. The ORFs are absent or truncated in the China/GD/2019 genome, with even additional genes adjacent to these areas (MGF 360 and MGF 110) deleted or truncated. According to previous observations, the ASFV BA71V strain isolated by repeated tissue culture would lead to the loss of MGF 110 family members [33]. Those ORFs still present may have a crucial role for replication in macrophages and virulence, but quite a few of them are still mostly uncharacterized. At present, ASFVs with MGF 505 and 360 genes deletion have been identified as the most promising vaccine candidates [34]. China/GD/2019 has a lack of MGF 360 family genes, which may be related to the virulence but not the infectivity of the virus. However, China/GD/2019 may not be attenuated in this way since other genes also can affect the virulence, and whether the missing genes in China/GD/2019 strain are due to frameshifts/single SNPs or full deletions needs further study. Comparing China/GD/2019 with China/2018/AnhuiXCGQ, a deletion of approximately 1 kb was found in China/GD/2019 which was located at the EP153R and EP402R genes. This deletion may cause changes in virus virulence and infectivity. As for the influence of sequencing artefacts, it is difficult to precisely distinguish between true low frequency variants and mutations and sequencing artefacts. Low template copies may be associated with higher probability of artefacts. There are many strategies to minimize the occurrence of sequencing artefacts, such as improving sequencing depth and template copies of samples, performing duplicate reactions for the same sample and so on.

This study demonstrates that genotype II ASFV circulating in Southern China (China/GD/2019) is genetically diverse. Further research is required to compare the whole ASFV genomes of genotype II from pigs in China with entire genome sequences of isolates from recent outbreaks to provide more insights into the genetic characterization and variation of ASFV.

## 4. Materials and Methods

### 4.1. Ethics Approval and Consent to Participate

We obtained written informed consent to collect clinical samples from the pig farm. All clinical samples collection was approved by the Institutional Animal Care and Use Committee of SunYat-sen University of China.

### 4.2. Field Samples

Clinical blood samples were collected from a pig farm of the Guangdong province in Southern China in 2019, and then were confirmed by real-time PCR with amplification targeting the B646L (p72) gene, with Ct values ranging from 15 to 23. Blood sample (Ct value = 15) collected from one pig which showed severe clinical symptoms was used for genome sequencing.

### 4.3. DNA Extraction

The field ASFV-positive blood samples were used to extract DNA for the next generation sequencing. Total DNA was extracted in duplicate using the QIAamp MinElute Virus Spin Kit (Qiagen) according to the protocol. The extraction kit retains both RNA and DNA, and a diagnostic conventional real-time PCR confirmed ASFV positive in the samples. The final elution volume was 30 μL of sterile nuclease-free water. 

### 4.4. Genome Sequencing and Assembly Analysis

For full genome sequencing, the extracted DNA was fragmented into a length of about 350 bp by Covaris ultrasonic processor, and then the DNA fragments were processed using the NeBNext ^®^Ultra™ DNA Library Prep Kit for Illumina (NEB, Ipswich, MA, USA) according to the manufacturer’s instructions. After quantification by Qubit 2.0 equipment, the DNA samples were sequenced using an Illumina NovaSeq PE150 sequencer (Illumina, San Diego, CA, USA). Raw reads were cleaned by filtering the inferior quality reads by Readfq v10. Swine genome (Sus scrofa 11.1, GenBank accession number GCF_000003025.6) reads were removed to eliminate host DNA contaminations. The viral genome was assembled using China/2018/SY-18 genome as a reference (GenBank accession number MH766894) by CLC Genomics Workbench v9. The genome sequence data generated in this study are available in GenBank database (accession number MW361944).

### 4.5. SNP/InDel Analysis

The global alignment between each sample and the reference sequence was carried out using the MUMmer (version 3.23) comparison software. Sequences of 100 bp on each side of the reference sequence SNP sites were extracted, and then BLAT software was used to compare the extracted sequences with the assembly results to verify the SNP sites. If the length of comparison is less than 101 bp, it is considered to be an untrustworthy SNP and will be removed. If the SNP is considered to be a repeating region after comparison for many times, it will also be removed. Finally, BLAST, TRF and Repeat mask software were used to predict the repeating sequence region of the reference sequence, and the SNP located in the repeating region was filtered, so that we could end up with a reliable SNP.

Insertion and deletion (InDel) refers to the insertion and deletion sequences of small segments of the genome. LASTZ software (Version 1.03.54) was used to compare the sample with the reference sequence, and then the comparison results were processed by axt_correction, axtSort and axtBest procedures to select the best comparison results, and the preliminary InDel results were obtained. Then, the upstream and downstream 150 bp (3xSD) of the reference sequence InDel site were selected and compared with the sequenced Reads of the sample using BWA software and SamTools for verification. Filtering yields reliable InDel.

### 4.6. Core Genes and Pan Genes Analysis

The common genes existing in all strains are called core genes. In addition to the core genes, other non-common genes are called dispensable genes. Specific genes only exist in a certain strain [35]. All dispensable genes and core genes are merged into pan genes. Core and pan genes analysis were performed using cd-hit (Version 4.6.1) software to cluster the protein sequences of multiple strains to be analyzed and mapped with R (Version 3.2.4). By comparing the gene/protein sequences of the different strains, we constructed core and pan genes tree of all strains.

### 4.7. Phylogenetic Analysis

Phylogenetic analysis of ASFV p72 (B646L) and p54 (E183L) genes was constructed based on strains for which these two genes were available from GenBank. Clustal W alignments were used for the alignment of the p72 and p54 nucleotide sequence.

The SNP phylogenetic tree was constructed based on SNP matrix of strains and reference strain population. For each strain, all SNPs were connected in the same order to obtain the same length of FASTA format (one of which is a reference sequence). The phylogenetic tree was constructed by maximum-likelihood (ML) method of Neighbor-Joining (NJ) method by TreeBeST software. The GenBank accession number, the year, genotype, reference and origin of ASFV genome sequences are listed in Table 1.

## 5. Conclusions

This study investigated the genomic characterization of an ASF outbreak in 2019 in Guangdong province, China. Genetic analysis indicates that the China/GD/2019 strain, which has new variations, is closely related to the genotype II ASFV isolates. Although belonging to genotype II, the ASFVs associated with the outbreaks in the Northern provinces of China have genetic diversity, and these outbreaks are correlated. Phylogenetic tree and comparative genomic analysis in this study will have multiple applications to improve our understanding of the degree of genetic evolution and variation differences between different isolates. This study provides useful information for exploring key factor of ASFV vaccine development.

## Figures and Tables

**Table 1:** ASFV strains used in this study.

Name	GenBank No.	Origin	Year	p72 gt	Reference
China/GD/2019	MW361944	China	2019	II	This study
China/2018/AnhuiXCGQ	MK128995	China	2018	II	Bao, J. et al. (2019)
POL/2015/Podlaskie	MH681419	Poland	2015	II	Olesen et al. (2018)
Georgia 2007/1	FR682468	Georgia	2007	II	Chapman et al. (2011)
Estonia 2014	LS478113	Estonia	2014	II	Zani et al. (2018)
Russia/Odintsovo_02/14	KP843857	Russia	2014	II	Unpublished
Spain/E75	FN557520	Spain	1975	I	de Villiers et al. (2010)
Malawi/Tengani 62	AY261364	Malawi	1962	V/I	Pan (1992)
SA/Pretorisuskop/96/4	AY261363	South Africa	1996	XX/I	Zsak et al. (2005)
Malawi/tick/Lil-20/1/1983	AY261361	Malawi	1983	VIII	Haresnape and Wilkinson (1989)
Portugal/OURT88/3	AM712240	Portugal	1988	I	Chapman et al. (2008)
Benin 97/1	AM712239	Benin	1997	I	Chapman et al. (2008)
Kenya/Ken06.Bus	KM111295	Kenya	2006	IX	Bishop et al. (2015)
Portugal/L60	KM262844	Portugal	1960	I	Portugal et al. (2015)
Portugal/NHV/1968	KM262845	Portugal	1968	I	Portugal et al. (2015)
Italy/47/Ss/2008	KX354450	Italy	2008	I	Granberg et al. (2016)
Spain/BA71	KP055815	Spain	1971	I	Rodriguez et al. (2015)
Uganda/R35/2015	MH025920	Uganda	2015	IX	Unpublished
China/Pig/HLJ/2018	MK333180	China	2018	II	Wen, X. et al. (2019)
China/DB/LN/2018	MK333181	China	2018	II	Wen, X. et al. (2019)
Georgia_2008/1	MH910495	Georgia	2008	II	Farlow et al. (2018)
Georgia_2008/2	MH910496	Georgia	2008	II	Farlow et al. (2018)
China/2018/SY-18	MH766894	China	2018	II	Miao, F. et al. (2018)
Pol16_20186_o7	MG939583	Poland	2016	II	Wozniakowski et al. (2018)
Pol16_29413_o23	MG939586	Poland	2016	II	Wozniakowski et al. (2018)
Pol17_04461_C210	MG939588	Poland	2017	II	Wozniakowski et al. (2018)
Pol17_05838_C220	MG939589	Poland	2017	II	Wozniakowski et al. (2018)
Russia/Kashino_04/13	KJ747406	Russia	2014	II	Vlasova et al. (2014)
ASFV_Belgium_2018/1	LR536725	Belgium	2018	II	Forth et al. (2019)
Pol16_20186_o7	MG939583	Poland	2018	II	Wozniakowski et al. (2018)
Spain/BA71V	NC_00165	Spain	1971	I	Yanez et al. (1995)

**Table 2:** Genome sequence variation between the China/GD/2019 strain and other representative genotype II strains.

Variation Type	China/2018/AnhuiXCGQ	China/Pig/HLJ/2018	China/DB/LN/2018	Pol16_20186_o7	Pol16_29413_o23	Pol17_05838_C220	Estonia 2014	Belgium_2018/1
Synonymous	1	1	1	1	1	1	63	1
Nonsynonymous	1	1	1	4	4	3	102	2
Intergenic	6	4	4	4	4	4	4	4
Deletion	0	1	1	0	0	0	0	0
Premature-stop	0	0	0	0	0	0	2	0
Frameshift mutation	0	1	1	0	0	0	0	0

**Table 3:** Genome sequence SNPs between the China/GD/2019 strain and other representative genotype II strains.

Type	Name	China/2018/ AnhuiXCGQ	China/Pig/ HLJ/2018	China/DB/ LN/2018	Pol16_20186 _o7	Pol16_29413 _o23	Pol17_05838 _C220	Belgium_2018 /1	China/GD/2019
Nonsyn	MGF_360 -6L	T	T	T	T	T	T	T	G
	D117L	C	C	C	C	C	C	T	C
	MGF_505 -4R	G	G	G	A	A	A	G	G
	K145R	C	C	C	A	A	A	C	C
	E199L	C	C	C	T	C	C	C	C
	E184L	C	C	C	C	T	C	C	C
Syn	F317L	A	A	A	A	A	A	A	T

Note. Nonsyn: Nonsynonymous; Syn: Synonymous.

## Data Availability

All data analyzed during this study are included in this published article. The raw data are available from the corresponding author upon reasonable request.

## Supplementary Materials

The following are available online at https://www.mdpi.com/article/10.3390/pathogens11010097/s1, Figure S1: Genome comparison between China/GD/2019 and China/2018/AnhuiXCGQ by collinearity analysis; Table S1: ORFs identity of core genes, dispensable genes and pan genes of 28 ASFV strains with China/GD/2019; Table S2: SNPs distribution of China/GD/2019 compared to Estonia 2014; Table S3: SNPs distribution of China/GD/2019 compared to 7 genotype II strains.

## References

[1] Enjuanes L., Carrascosa A.L., Viñuela E. (1976). Isolation and properties of the DNA of African swine fever (ASF) virus. J. Gen. Virol..

[2] Dixon L.K. (1988). Molecular cloning and restriction enzyme mapping of an African swine fever virus isolate from Malawi. J. Gen. Virol..

[3] Galindo I., Alonso C. (2017). African Swine Fever Virus: A Review. Viruses.

[4] Martins C.L., Leitão A.C. (1994). Porcine immune responses to African swine fever virus (ASFV) infection. Vet. Immunol. Immunopathol..

[5] Ortín J., Enjuanes L., Viñuela E. (1979). Cross-links in African swine fever virus DNA. J. Virol..

[6] Costard S., Wieland B., de Glanville W., Jori F., Rowlands R., Vosloo W., Roger F., Pfeiffer D.U., Dixon L.K. (2009). African swine fever: How can global spread be prevented?. Philos. Trans. R. Soc. Lond. Ser. Biol. Sci..

[7] Li L., Ren Z., Wang Q., Ge S., Liu Y., Liu C., Liu F., Hu Y., Li J., Bao J. (2019). Infection of African swine fever in wild boar, China, 2018. Transbound. Emerg. Dis..

[8] Bao J., Wang Q., Lin P., Liu C., Li L., Wu X., Chi T., Xu T., Ge S., Liu Y. (2019). Genome comparison of African swine fever virus China/2018/AnhuiXCGQ strain and related European p72 Genotype II strains. Transbound. Emerg. Dis..

[9] Zhao D., Liu R., Zhang X., Li F., Wang J., Zhang J., Liu X., Wang L., Zhang J., Wu X. (2019). Replication and virulence in pigs of the first African swine fever virus isolated in China. Emerg. Microbes. Infect..

[10] Zhou X., Li N., Luo Y., Liu Y., Miao F., Chen T., Zhang S., Cao P., Li X., Tian K. (2018). Emergence of African Swine Fever in China, 2018. Transbound. Emerg. Dis..

[11] Yáñez R.J., Rodríguez J.M., Nogal M.L., Yuste L., Enríquez C., Rodriguez J.F., Viñuela E. (1995). Analysis of the complete nucleotide sequence of African swine fever virus. Virology.

[12] Blasco R., de la Vega I., Almazán F., Agüero M., Viñuela E. (1989). Genetic variation of African swine fever virus: Variable regions near the ends of the viral DNA. Virology.

[13] Dixon L.K., Twigg S.R., Baylis S.A., Vydelingum S., Bristow C., Hammond J.M., Smith G.L. (1994). Nucleotide sequence of a 55 kbp region from the right end of the genome of a pathogenic African swine fever virus isolate (Malawi LIL20/1). J. Gen. Virol..

[14] Blasco R., Agüero M., Almendral J.M., Viñuela E. (1989). Variable and constant regions in African swine fever virus DNA. Virology.

[15] de la Vega I., Viñuela E., Blasco R. (1990). Genetic variation and multigene families in African swine fever virus. Virology.

[16] Vydelingum S., Baylis S.A., Bristow C., Smith G.L., Dixon L.K. (1993). Duplicated genes within the variable right end of the genome of a pathogenic isolate of African swine fever virus. J. Gen. Virol..

[17] Zhu Z., Chen H., Liu L., Cao Y., Jiang T., Zou Y., Peng Y. (2020). Classification and characterization of multigene family proteins of African swine fever viruses. Brief. Bioinform..

[18] Gallardo C., Fernández-Pinero J., Pelayo V., Gazaev I., Markowska-Daniel I., Pridotkas G., Nieto R., Fernández-Pacheco P., Bokhan S., Nevolko O. (2014). Genetic variation among African swine fever genotype II viruses, eastern and central Europe. Emerg. Infect..

[19] Simulundu E., Sinkala Y., Chambaro H.M., Chinyemba A., Banda F., Mooya L.E., Ndebe J., Chitanga S., Makungu C., Munthali G. (2018). Genetic characterisation of African swine fever virus from 2017 outbreaks in Zambia: Identification of p72 genotype II variants in domestic pigs. Onderstepoort J. Vet. Res..

[20] Wen X., He X., Zhang X., Zhang X., Liu L., Guan Y., Zhang Y., Bu Z. (2019). Genome sequences derived from pig and dried blood pig feed samples provide important insights into the transmission of African swine fever virus in China in 2018. Emerg. Microbes Infect..

[21] Tao D., Sun D., Liu Y., Wei S., Yang Z., An T., Shan F., Chen Z., Liu J. (2020). One year of African swine fever outbreak in China. Acta Trop..

[22] Bastos A.D., Penrith M.L., Crucière C., Edrich J.L., Hutchings G., Roger F., Couacy-Hymann E.G., Thomson G.R. (2003). Genotyping field strains of African swine fever virus by partial p72 gene characterisation. Arch. Virol..

[23] Iyer L.M., Balaji S., Koonin E.V., Aravind L. (2006). Evolutionary genomics of nucleo-cytoplasmic large DNA viruses. Virus Res..

[24] Yu M., Morrissy C.J., Westbury H.A. (1996). Strong sequence conservation of African swine fever virus p72 protein provides the molecular basis for its antigenic stability. Arch. Virol..

[25] Irusta P.M., Borca M.V., Kutish G.F., Lu Z., Caler E., Carrillo C., Rock D.L. (1996). Amino acid tandem repeats within a late viral gene define the central variable region of African swine fever virus. Virology.

[26] Boinas F.S., Hutchings G.H., Dixon L.K., Wilkinson P.J. (2004). Characterization of pathogenic and non-pathogenic African swine fever virus isolates from Ornithodoros erraticus inhabiting pig premises in Portugal. J. Gen. Virol..

[27] Chapman D.A.G., Tcherepanov V., Upton C., Dixon L.K. (2008). Comparison of the genome sequences of non-pathogenic and pathogenic African swine fever virus isolates. J. Gen. Virol..

[28] Kipanyula M.J., Nong′ona S.W. (2017). Variations in clinical presentation and anatomical distribution of gross lesions of African swine fever in domestic pigs in the southern highlands of Tanzania: A field experience. Trop. Anim. Health Prod..

[29] de Villiers E.P., Gallardo C., Arias M., da Silva M., Upton C., Martin R., Bishop R.P. (2010). Phylogenomic analysis of 11 complete African swine fever virus genome sequences. Virology.

[30] Farlow J., Donduashvili M., Kokhreidze M., Kotorashvili A., Vepkhvadze N.G., Kotaria N., Gulbani A. (2018). Intra-epidemic genome variation in highly pathogenic African swine fever virus (ASFV) from the country of Georgia. Virol. J..

[31] Owolodun O.A., Obishakin E.T., Ekong P.S., Yakubu B. (2010). Investigation of African swine fever in slaughtered pigs, Plateau state, Nigeria, 2004-2006. Trop. Anim. Health Prod..

[32] Phologane S.B., Bastos A.D., Penrith M.L. (2005). Intra- and inter-genotypic size variation in the central variable region of the 9RL open reading frame of diverse African swine fever viruses. Virus. Genes.

[33] Pan I.C. (1992). African swine fever virus: Generation of subpopulations with altered immunogenicity and virulence following passage in cell cultures. J. Vet. Med. Sci..

[34] Afonso C.L., Piccone M.E., Zaffuto K.M., Neilan J., Kutish G.F., Lu Z., Balinsky C.A., Gibb T.R., Bean T.J., Zsak L. (2004). African swine fever virus multigene family 360 and 530 genes affect host interferon response. J. Virol..

[35] Wang L., Luo Y., Zhao Y., Gao G.F., Bi Y., Qiu H.J. (2020). Comparative genomic analysis reveals an ’open’ pan-genome of African swine fever virus. Transbound. Emerg. Dis..

