# Supplementary material for: Genetic Characterization and Variation of African Swine Fever Virus China/GD/2019 Strain in Domestic Pigs
Source: Pathogens. 2022 Jan 14;11(1):97. doi: 10.3390/pathogens11010097 (PMC8780551; doi:10.3390/pathogens11010097)
Supplement: Supplementary file 1 [file pathogens-11-00097-s001.zip › Supplementary Files/Supplementary Figure S1.pdf]

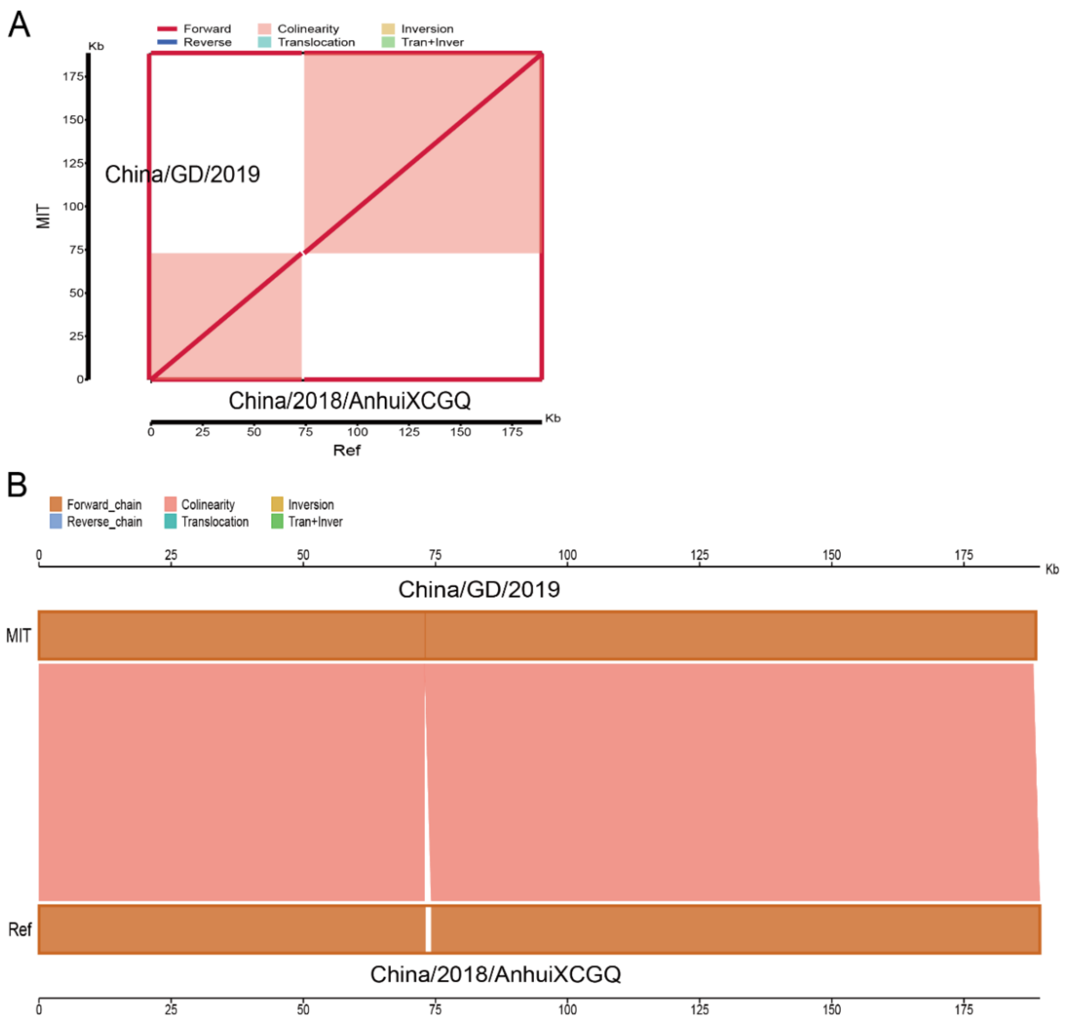

Figure S1. Genome comparison between China/GD/2019 and China/2018/AnhuiXCGQ: A deletion of approximately 1kb was found in China/GD/2019 genome which located at EP153R and EP402R genes in comparison to China/2018/AnhuiXCGQ strain. (A) Collinearity analysis of China/GD/2019 with China/2018/AnhuiXCGQ. (B) Comparative genomic analysis of China/GD/2019 and China/2018/AnhuiXCGQ.
